# Whole-Genome Sequencing and Intestinal Metagenome Sequencing Revealed the Carriage and Transmission of *Salmonella enterica* in Xinjiang, China

**DOI:** 10.3390/pathogens15090930

**Published:** 2026-09-03

**Authors:** Jia Huang, Hongqing Zhao, Fulong Wang, Beini Zeng, Zichao Ma, Rong Wang, Yang Yang, Muhetaer Amier, Bairena Yilihaer, Yaoqin Lu

**Affiliations:** 1 Institute of Pathogenic Biological Detection, Xinjiang Center for Disease Control and Prevention, Urumqi 830000, China; huangjia1234562021@126.com (J.H.); 17794815998@163.com (F.W.); beinisdu@163.com (B.Z.); wrong202607@163.com (R.W.); 18399685519@163.com (Y.Y.); 15622122417@163.com (M.A.); 2 Administration Office, Institute of Infectious Disease Prevention and Control, Chinese Center for Disease Control and Prevention, Beijing 102206, China; zhaohq@chinacdc.cn; 3 Research and Technology Transfer Department, Xinjiang Center for Disease Control and Prevention, Urumqi 830000, China; m363063121@126.com; 4 Public Health College, Xinjiang Medical University, Urumqi 830000, China; 19999211160@163.com

**Keywords:** whole-genome sequencing, metagenome sequencing, gut, *Salmonella*, healthy people, Xinjiang, China

## Abstract

**Background:** *Salmonella enterica*, a major foodborne pathogen, poses a severe global public health threat. However, data regarding its carriage characteristics, transmission patterns, and association with intestinal microbiota in healthy populations of Xinjiang, China, remain insufficient, limiting the formulation of targeted salmonellosis prevention and control strategies. **Methods:** In this study, 31 *Salmonella enterica* strains isolated from more than 2000 healthy individuals in Urumqi were subjected to whole-genome sequencing to analyze serovars, antimicrobial resistance (AMR) genes, and virulence genes. Meanwhile, metagenomic sequencing was performed on 50 fecal samples (culture negative) from 2000 healthy individuals to investigate intestinal microbiota structure, *Salmonella enterica* prevalence, and related microbial taxa. **Results:** The results showed that 60% of samples (30/50) were positive by the read-based criterion (≥1000 *Salmonella*-specific reads), while the assembly-verified criterion (≥1000 reads and contigs > 1 kb) confirmed *Salmonella*-specific sequences in 12% (6/50). Among the 31 culture-confirmed isolates, *Salmonella* Typhimurium and *Salmonella* Paratyphi B were the dominant serovars, together accounting for 60%. All isolates harbored core virulence genes for Type III secretion system and adhesion factors, with low AMR gene carriage and no multidrug-resistant strains. Phylogenetic analysis showed that Urumqi-derived isolates were distributed across multiple genomic clusters, suggesting active inter-regional circulation of S. enterica within the available dataset. *Salmonella enterica* carriage did not affect gut microbial α-diversity but altered community composition, with *Escherichia coli*, *Shigella flexneri*, and *Klebsiella pneumoniae* as key associated taxa. **Conclusions:** This study found that Urumqi-derived isolates are widely distributed across genomic clusters in Xinjiang, with a unique local transmission chain identified, though definitive source attribution requires further geographically balanced sampling. *Salmonella enterica* carriage exhibited ecological niche synergy with intestinal *Enterobacteriaceae*, but did not significantly affect gut microbial diversity.

## 1. Introduction

*Salmonella enterica*, a Gram-negative, facultatively anaerobic, non-sporulating rod-shaped bacterium of the family Enterobacteriaceae, is a leading cause of salmonellosis worldwide and a major foodborne pathogen responsible for numerous global foodborne outbreaks. Globally, nontyphoidal Salmonella (NTS) alone is estimated to cause approximately 93.8 million cases of gastroenteritis and 155,000 deaths annually, of which around 85% are attributable to contaminated food, representing the dominant subset of foodborne salmonellosis [1]. The substantial morbidity, mortality, and healthcare costs associated with foodborne salmonellosis impose a heavy public health and economic burden worldwide [1]. *Salmonella enterica* is classified into typhoidal and nontyphoidal (NTS) serovars. Typhoidal serovars, such as *Salmonella* Typhi and *Salmonella* Paratyphi A, cause typhoid and paratyphoid fever. Severe cases may lead to neurological symptoms, leukopenia, septicemia, or even death. NTS serovars (e.g., *Salmonella Typhimurium* and *Salmonella Enteritidis*) usually induce mild to severe gastroenteritis, with rare bacteremia. Clinically, salmonellosis shows three main manifestations: gastroenteritis, septicemia, and enteric (typhoid) fevers [2]. The pathogenesis of *Salmonella enterica* involves multiple steps and mechanisms, including invasion of non-phagocytic host cells via SPI-mediated intestinal attack (particularly SPI-1-encoded T3SS), formation of *Salmonella*-containing vacuoles (SCVs) that support intracellular survival and replication, regulation of inflammatory pathways and autophagy, evasion of adaptive immunity through interactions with immune cells, and dissemination through the reticuloendothelial system (RES) in macrophages [3].

Antimicrobial resistance (AMR) adds a further critical dimension to this burden, distinct from overall disease incidence. Multidrug-resistant (MDR) strains compromise treatment efficacy and have been isolated from diverse sources including seafood; resistance to ampicillin, chloramphenicol, tetracycline, and nalidixic acid are commonly reported. Currently, antimicrobial-resistant bacterial infections (including resistant *Salmonella*) are estimated to cause approximately 700,000 deaths globally each year, and this figure is projected to rise to 10 million deaths annually by 2050, accompanied by enormous economic losses [4]. Vulnerable populations, including young children and elderly people with weak immune systems, are disproportionately affected [5,6]. *Salmonella enterica* is transmitted through contaminated food (including seafood such as oysters and shrimp) and water. Environmental factors, including temperature, precipitation, pH, and nutrient availability, influence its growth and transmission dynamics [7]. Effective infection control measures include improving food hygiene, ensuring water safety, limiting antibiotic use in food animals, and strengthening public health cooperation.

As a major foodborne pathogen, *Salmonella enterica* is transmitted along the “farm-to-fork” chain [8]. Food accounts for approximately 85% of human infections, with poultry, red meat, seafood, and fruits and vegetables as key sources [9]. The environment, pets, wild animals, and long-term survival on surfaces with biofilm formation further boosts its spread [10]. For human carriage, 1–6% of typhoid-infected patients become chronic carriers, and carriage rates among food handlers vary greatly by region [11]. In China, nearly half of carried strains are typhoidal serovars [12]. Two subpopulations of *Salmonella enterica* cooperate in the gut to promote transmission [13]. Given its diverse transmission routes, region-targeted control is essential. Further studies on environmental factors and global surveillance, especially for high-risk groups, are needed for better prevention.

Whole-genome sequencing (WGS) is a powerful tool widely used throughout the food industry chain. It has been applied to trace *Salmonella enterica* transmission in meat production [14], kitchen cross-contamination [15], dairy supply chains [16], and cross-regional outbreaks [12], supporting effective source tracking and supervision [17]. Beyond traceability, WGS enables high-precision prediction of antimicrobial resistance in *Salmonella enterica* and monitors transmission dynamics and AMR gene transfer [18]. Complemented by metagenomics and metabolic network analysis, microbial interactions can be further elucidated. For instance, cooperation between *Salmonella* Typhimurium and *Escherichia coli* can enhance the antibiotic tolerance of the pathogen, providing a more comprehensive understanding of *Salmonella enterica* survival and transmission mechanisms [19].

Whole metagenome sequencing (WMS), as a culture-independent approach, serves a crucial role in foodborne outbreak investigations: it detected outbreak-causing S. Heidelberg in stool samples, revealed gut microbiota alterations and co-infections [20], and analyzed AMR gene distributions in complex environments such as dairy and beef production facilities [21]. WMS offers distinct advantages, including high resolution, comprehensive data analysis capabilities, and strong cross-linkage potential. However, its results require integration with epidemiological data and food traceability records, alongside standardized sequencing and bioinformatics analysis protocols, to maximize their utility.

Given the vast territory and uneven public health resources across Xinjiang, we designed this study as a pilot phase of the province-wide *Salmonella* surveillance program. We collected clinical *Salmonella enterica* isolates from multiple prefectures to characterize the genomic landscape of circulating strains, and selected Urumqi, the provincial capital with the highest population density and most complete public health monitoring system, as the pilot site for the metagenomic investigation of asymptomatic intestinal carriage. In this study, 31 *Salmonella enterica* strains were isolated from healthy participants in Urumqi, Xinjiang, China. WGS was used to determine the serovars, antimicrobial resistance genes, and virulence genes of these 31 strains. Additionally, 50 fecal samples were randomly selected from 2000 healthy participants for metagenomic sequencing, to analyze differences in microbial communities, the prevalence of *Salmonella enterica* and other pathogenic bacteria, and their associated characteristic microbes. These analyses clarify the transmission characteristics of *Salmonella enterica* and the microbial community profiles of *Salmonella enterica* carriers in Urumqi, Xinjiang, thereby providing theoretical support for the prevention and control of *Salmonella*-induced diseases.

## 2. Material and Methods

For the investigation of asymptomatic *Salmonella enterica* carriage, we recruited healthy participants from Urumqi as a pilot cohort. Urumqi was selected as the study site for three main reasons: (1) it is the political, economic, and transportation hub of Xinjiang, with the largest cross-regional population flow and highest risk of foodborne pathogen transmission; (2) it has a well-established community public health monitoring network, which ensures standardized sample collection, cold-chain transportation, and quality control; (3) as a pilot study of metagenomics-based carriage surveillance, a single-site design helps to control confounding factors and verify the stability of the analytical pipeline. A total of 2000 healthy individuals were enrolled for traditional culture screening, and 50 culture-negative samples were randomly selected for metagenomic sequencing.

### 2.1. Study Population

This cross-sectional study was conducted in Urumqi, Xinjiang, China. All *Salmonella enterica* isolates were derived from a single healthy carrier screening cohort, with participants recruited from routine physical examination populations at local medical centers. Healthy status was confirmed by standardized criteria: eligible participants aged 18–75 years had no acute gastrointestinal symptoms at sampling, no history of enteric infection in the preceding 3 months, no chronic gastrointestinal disorders, no antibiotic or gastrointestinal medication use within 1 month, and no immunocompromised conditions. These exclusion criteria were applied to ensure a genuinely healthy study population and to minimize confounding, as recent enteric infection, chronic gastrointestinal disorders, and antibiotic or gastrointestinal medication use can substantially alter the gut microbiota composition and *Salmonella* carriage status, thereby confounding the interpretation of carriage-associated microbial signatures. A total of more than 2000 eligible individuals were enrolled and sampled from Hospital of Traditional Chinese Medicine Affiliated to Xinjiang Medical University, from whom 31 non-duplicate *S. enterica* isolates were recovered from stool specimens and subjected to whole-genome sequencing and subsequent bioinformatic analysis (Table S2).

### 2.2. Sample Collection and Bacterial Isolation

In this study, both bacterial isolation and metagenomic sequencing samples were derived from a single healthy carrier screening cohort in Urumqi, Xinjiang. *Salmonella* isolates were obtained from culture-positive healthy individuals, while 50 fecal samples for metagenomic sequencing were randomly selected from the same cohort of over 2000 healthy individuals using the sample() function in R (v4.3.0) [22]. The isolation of *Salmonella enterica* was carried out according to the established cost-effective protocol for clinical laboratories [23]. Stool samples were collected by participants into sterile, disposable, screw-cap containers following standardized instructions to avoid contact with urine or water. Each container was immediately sealed after collection and transported to the laboratory under cold-chain conditions (4 °C) within 2 h, or stored at 4 °C for no more than 24 h before processing. All sample handling was performed in a biosafety cabinet using sterile consumables to prevent cross-contamination. Samples were inoculated onto selective media including *Salmonella* chromogenic medium (CHROMagar, Paris France) and *Salmonella*–*Shigella* (SS) agar (Oxoid, Basingstoke UK), and then incubated at 35–37 °C for 18–24 h. Presumptive *Salmonella* colonies were first identified at the species level using a panel of biochemical tests, including triple sugar iron (TSI) agar, urease, motility, indole, and citrate utilization tests (or API 20E strips). Isolates biochemically confirmed as *Salmonella* were then serotyped by slide agglutination assays using commercially available O- and H-antigen antisera (SSI Diagnostica, Copenhagen Denmark), following the Kauffmann–White scheme. For each sample, one to three representative presumptive colonies were purified and subjected to biochemical and serological confirmation; a single confirmed isolate per sample was retained for whole-genome sequencing. Selenite enrichment broth (BD Difco, Franklin Lakes, NJ, USA) combined with xylose–lysine–deoxycholate (XLD) agar (Oxoid, Basingstoke UK) was used to further improve isolation efficiency. Single colonies from each sample were selected, and purified *Salmonella* isolates were stored on dry ice before being sent to Hangzhou Baiyi Technology Co., Ltd., for whole-genome sequencing and analysis. Fecal samples reserved for metagenomic sequencing were stored at −80 °C.

### 2.3. Whole-Genomic Sequencing Data Analysis

Total DNA was divided into two aliquots for sequencing by a NovaSeq 6000 (Illumina, San Diego, CA, USA) with paired-end 150 bp reads and a GridION (Nanopore, Oxford UK) with long reads. The sequencing data were then processed by Pathogenic Whole Genome Analysis System V1.0 on BAIYITECH Pathogenic Microorganism Bioinformatic Analysis Platform v6.0 (Hangzhou Baiyi Technology Co., Ltd., Hangzhou China). The genome analysis pipeline consists of the following steps: First, the data are quality controlled and filtered using fastQC (v0.12.1) [24] and fastp (v1.01) [25]. The de novo hybrid assembly was performed using Unicycler software (v.0.5.0) [26] for long reads and short reads. The contig was then polished 3 times by medaka (v2.1.0) (Nanopore, Oxford UK) to optimize assembly results and improve sequence accuracy based on short reads. Kraken2 (v1.2) [27] was used for species classification, combined with Bracken (v2.8) [28] for abundance correction. Core genome multilocus sequence typing (cgMLST) was performed using cgMLSTFinder based on the KMA algorithm [29] against the *Salmonella* cgMLST schema, following the gene-by-gene approach described by Maiden et al. [30]. The serovars of *Salmonella enterica* were also analyzed by sistr (v1.1.3) [31]. For phylogenetic and transmission cluster analyses, 181 additional *Salmonella enterica* isolates were included from 14 prefectures and 29 districts across Xinjiang (China National Pathogen Identification Network, CPIN). These isolates were collected between March 2021 and February 2025 from clinical specimens and environmental samples. For population genomic analysis of the *Salmonella enterica* isolates, whole-genome single nucleotide polymorphism (SNP) calling and core genome alignment were performed using the snippy pipeline (v4.6.0) with *Salmonella enterica* serovar Typhimurium LT2 (NC_003197.1) as the reference genome. SNP filtering criteria included a minimum read depth of 10×, minimum mapping quality of 20, and minimum base quality of 20. Heterozygous sites were excluded, and recombinant regions were detected and removed using Gubbins (v2.4.1). Pairwise SNP distances between all isolates were calculated using snp-dists, and pairwise genetic distances were further estimated under the Tamura–Nei 1993 (TN93) [32] nucleotide substitution model for evolutionary and population structure analyses. Based on the pairwise SNP distance matrix, a minimum spanning tree (MST) was constructed using GrapeTree (v1.5.0) [33] to visualize genetic relatedness and clustering patterns, with each node representing one isolate and color-coded by serotype. Isolates differing by ≤20 pairwise SNPs were defined as belonging to the same genetic cluster. All the software, except for those specifically stating the parameters to be used, used the default parameters set by the software.

### 2.4. Metagenomic Sequencing Data Analysis

Total DNA was sequenced using a NovaSeq 6000 (Illumina, San Diego, CA, USA) with paired-end 150 bp reads. Raw reads were quality-filtered and subsampled to an equal sequencing depth (rarefaction) across all samples prior to taxonomic classification, ensuring that *Salmonella*-specific read counts were comparable between samples and not confounded by differences in sequencing depth. Next, the subsampled sequencing data were processed by the Broad-Spectrum Pathogenic Microorganism and Antibiotic Resistance Analysis System V4.0 on BAIYITECH Pathogenic Microorganism Bioinformatic Analysis Platform v6.0 (Hangzhou Baiyi Technology Co., Ltd. Hangzhou China). Raw reads were removed adapters, the human reads by bowtie2 (v2.5.0) [34], and then quality-trimmed by fastqc (v0.12.1) [24]. The clean reads were assembled de novo by metaSPAdes (v3.15.4) [35] and megahit (v1.2.9) [36] with default parameters, were merged, and then de-duplicated by cd-hit (v4.8.1) [37]. The taxonomic composition was performed using Kraken2 (v1.2) [27] and adjusted twice by bracken (v2.8) [28] and aligning draft MAGs to the Clean Reads with the merged and de-duplicated contigs as bridge. To reduce false positives arising from homologous sequences among closely related Enterobacteriaceae (e.g., *Salmonella*, *E. coli*, *Shigella*), we applied a two-tier detection strategy. Samples were first screened by a read-based criterion (≥1000 *Salmonella*-specific reads). Those meeting this threshold were then subjected to de novo assembly, and samples with *Salmonella*-derived contigs > 1 kb were classified as assembly-verified positive. Accordingly, two criteria were defined: (i) the read-based criterion (≥1000 reads), and (ii) the assembly-verified criterion (≥1000 reads and contigs >1 kb). All downstream comparative analyses (α/β-diversity, random forest, and co-occurrence network) used the read-based positive group as the case definition.

Microbial co-occurrence network analysis was performed based on species abundance profiles by the microeco (v2.3.0) package [38] in R (v4.3.0) [22]. Absolute abundance data were first normalized to relative abundance using the decostand function in the vegan (v2.6.0) package [39] in R (v4.3.0) [22], and low-abundance taxa with a mean relative abundance below 0.01% were filtered out. Pairwise Spearman correlations were calculated between all retained taxa. The correlation threshold for edge retention was determined using the random matrix theory (RMT)-based approach, resulting in a cutoff of Spearman’s *r* ≥ 0.74 with *p* < 0.05. The resulting network contained 2221 nodes and 55,604 edges, and was visualized using Gephi (v0.9.7) [40]. Community (module) detection was performed using the Louvain algorithm [41], yielding a modularity of 0.679. Node roles were classified based on within-module connectivity (*z*-score) and among-module connectivity (participation coefficient, *c*) following Guimerà & Amaral (2005) [42] and Olesen et al. (2007) [43]: peripherals (*z* < 2.5, *c* < 0.62), connectors (*z* < 2.5, *c* ≥ 0.62), module hubs (*z* ≥ 2.5, *c* < 0.62), and network hubs (*z* ≥ 2.5, *c* ≥ 0.62).

### 2.5. Virulence Gene Profiling and Antimicrobial Resistance Gene Prediction

Virulence gene profiling was performed by aligning all predicted proteins against the Virulence Factor Database (VFDB) [44] to annotate virulence-associated genes for subsequent comparative analyses. Antimicrobial resistance genes were annotated for each genome by BLASTP search of all Prokka-predicted proteins against the Comprehensive Antibiotic Resistance Database (CARD, v 3.2.9) [45,46] using BLAST v2.11.0 [47]. Only hits classified by Resistance Gene Identifier (RGI, v6.0.3) [46] as “strict” or “perfect” and showing amino-acid identity ≥ 90% together with alignment coverage ≥ 80% (relative to the corresponding reference sequence in CARD) were retained as putative Antibiotic Resistance Genes (ARGs) [48]. For downstream analyses, retained hits were grouped according to their Antibiotic Resistance Ontology (ARO) annotations at the levels of antibiotic drug class and resistance mechanism/gene family. Heatmaps displaying virulence gene and antimicrobial resistance gene profiles were generated using the pheatmap package (v1.0.12) [49] in R (v4.3.0) [22] based on gene presence/absence data. Isolates were hierarchically clustered and annotated with serotype information via colored side bars.

### 2.6. Statistical Analysis

Principal coordinates analysis (PCoA) was conducted using the ade4 package (v1.7) [50] in R (v4.3.0) [22] to further illustrate the overall population structure and genetic differentiation across serogroups. Boxplots were generated to compare the counts of antimicrobial resistance genes and virulence genes among serotype groups, with statistical significance assessed by the Kruskal–Wallis test [51]. For metagenomic data from the healthy cohort, a random forest classification model was established using the random Forest package (v4.7) [52] in R based on microbial species abundance profiles, to identify key microbial biomarkers associated with *Salmonella* intestinal carriage. Variable importance was measured by the mean decrease in the Gini index to rank discriminatory taxa, and model performance was evaluated via 10-fold cross-validation, and model accuracy and area under the receiver operating characteristic curve (AUC) were reported. Statistical analyses were conducted using GraphPad Prism version 10 and R version 4.3.0. Data were summarized as the mean ± standard deviation (SD) or median with interquartile range (IQR), as appropriate. Group differences were assessed by the adonis test [38] (vegan package v2.6.0), with homogeneity of group dispersions verified by the beta-disper test [53]. Other group comparisons were assessed by *t*-test or non-parametric equivalents [54] as appropriate. For multiple comparisons, *p* values were adjusted using the Benjamini–Hochberg false discovery rate (FDR) [55] method, with an adjusted *p* value < 0.05 considered statistically significant. All statistical tests were two-sided.

## 3. Results

### 3.1. Genomic Features of Salmonella Enterica Isolates

Thirty-one *Salmonella enterica* isolates from healthy human subjects were sequenced and analyzed using second- and third-generation sequencing in this study. All isolates were obtained from healthy individuals, confirming that the corresponding genomes represent *S. enterica* strains capable of colonizing the healthy human host.

As summarized in Table S8, these genomes exhibited compact architecture and high overall compositional similarity. Genome sizes ranged from 4.51 to 4.95 Mb, with a median of ~4.76 Mb. GC content was tightly clustered at approximately 52% across most isolates, ranging from 52.11% to 52.29%. N50 values for most isolates were at or above 4.5 Mb, with the exception of SAL684, which had a comparatively lower N50 of 2.58 Mb. The L50 value was 1 for all genomes, demonstrating that over 50% of each assembly was contained within a single contig and confirming the high contiguity of these assemblies. Each genome encoded 4427 to 5015 predicted genes, including 4318 to 4903 protein-coding sequences (CDSs) and 84 to 90 tRNA genes. These genomic features were consistent with those reported for reference *S. enterica* genomes (e.g., *Salmonella* Typhimurium, with a genome size of ~4.70 Mb and GC content of ~52.12%), validating the suitability of this dataset for subsequent comparative and functional genomic analyses.

### 3.2. Virulence Gene Repertoire and Antimicrobial Resistance Genes Among the Isolates

**Figure 1:**
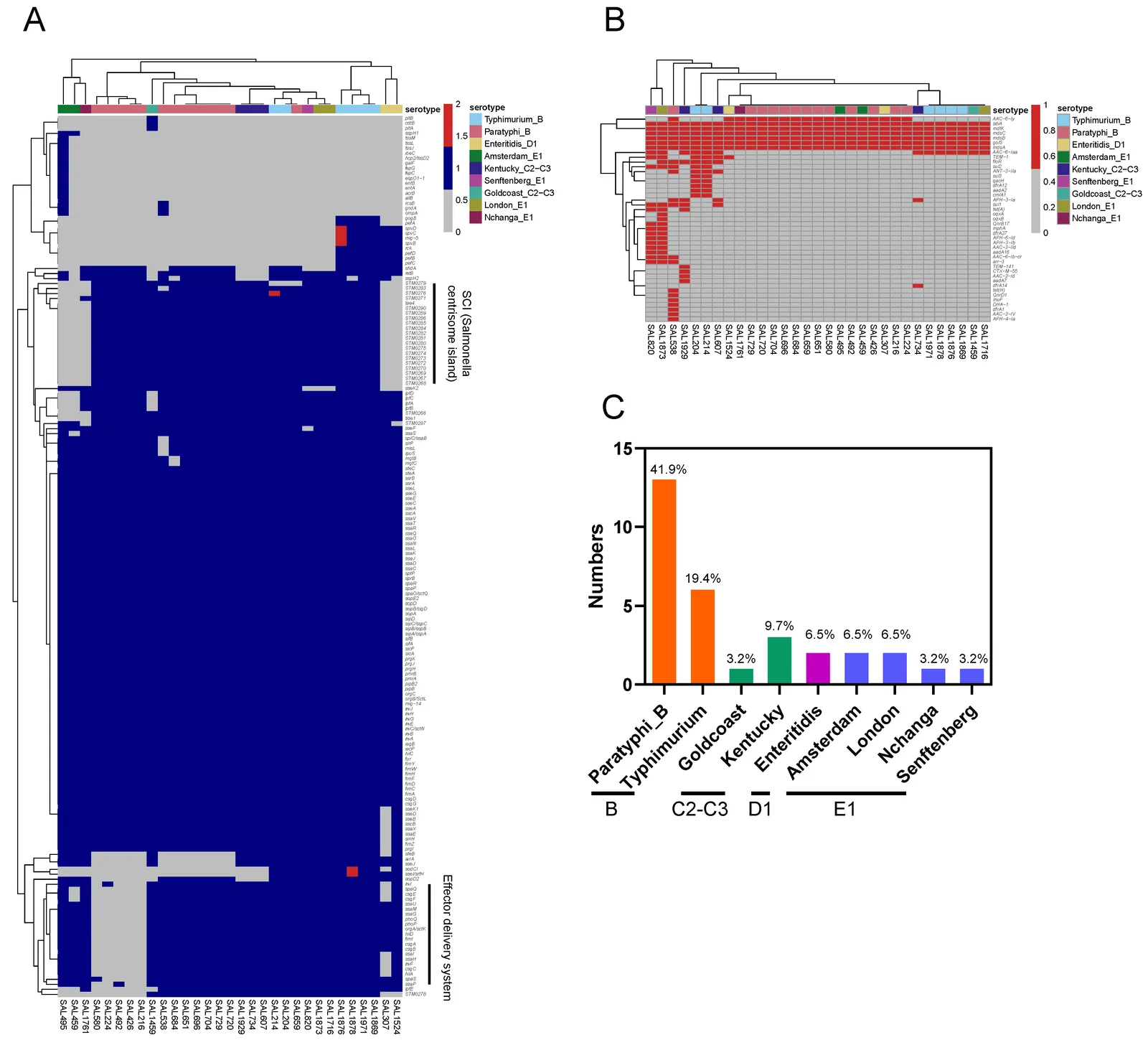
Distribution of virulence genes, drug resistance (DR) genes, and strain–gene associations in 31 healthy human-associated *Salmonella enterica* isolates. (**A**) Serovar-specific distribution of key virulence genes, presented as a heatmap. The y-axis represents the relative abundance of virulence genes, and the x-axis lists identified serovars. (**B**) Serovars-restricted DR gene carriage, presented as a heatmap. The y-axis represents the relative abundance of DR genes, and the x-axis lists the corresponding serovars. Gene names were simplified for visualization purposes (see Supplementary Table S1 for details). (**C**) Association of the 31 *S. enterica* isolates with virulent genes linked to effector delivery systems. The right panel shows the counts and percentages of isolates harboring virulent genes across distinct serovars and serogroups.

Virulence gene analysis of 31 *Salmonella enterica* isolates from healthy individuals (designated the SAL series) revealed that these strains belonged to 10 serovars, including *Salmonella* Typhimurium, *Salmonella* Paratyphi B, and *Salmonella* Enteritidis. Their virulence gene distribution exhibited a pattern of core conservation with serovar-specific divergence and was dominated by genes associated with effector delivery systems (41.9%; Figure 1A). All isolates harbored core components of the Type III secretion system (T3SS) encoded by Salmonella Pathogenicity Island 1 (SPI-1) and Salmonella Pathogenicity Islands 2 (SPI-2) (e.g., *invA*, *ssrA*, *sseL*), as well as genes encoding the Type VI secretion system (T6SS) from SPI-6 (e.g., STM0266–STM0290, *tae*4). Further, all isolates generally retained adherence-associated genes (e.g., *fimA*, *csgA*) and stress survival genes (e.g., *sodCI*), providing a molecular foundation for their intestinal colonization, immune evasion, and interbacterial competition in healthy human hosts. Distinct serovar-specific virulence gene signatures were identified: for example, the typhoid toxin subunit gene *pltB* was uniquely present in *Salmonella enterica* subsp. enterica serovar Goldcoast serovars; antimicrobial resistance-associated genes (*aac(6*′)-*Iy*, *aph(4)-Ia*) were predominantly distributed in *Salmonella* Paratyphi B, *Salmonella* Enteritidis and *Salmonella enterica* subsp. enterica serovar Amsterdam, with respective carriage rates of 19.4% and 9.7%; and regulatory genes including *mdtK* and *sdiA* were detected only in a small subset of strains. The composition and distribution profiles of these virulence genes demonstrate that *S. enterica* carried by healthy individuals, despite lacking large repertoires of highly toxic exotoxin genes (e.g., *cdtB*, *spvB*), retain the capacity for stable intestinal colonization via conserved effector delivery systems, adhesion factors, and a limited set of resistance-associated genes.

Furthermore, analysis of drug resistance (DR) genes in these 31 healthy human-associated *Salmonella enterica* isolates, representing 10 serovars (e.g., *Salmonella* Typhimurium, *Salmonella* Paratyphi B, *Salmonella* Goldcoast, *Salmonella* London), revealed a low-to-moderate DR gene carriage rate with distinct serovar-specific distribution patterns, as illustrated in Figure 1B. The detected DR genes primarily mediated resistance to aminoglycosides, fluoroquinolones, and multiple other drug classes. Specifically, the chromosomally encoded aminoglycoside acetyl-transferase gene *aac-(6*′)-*Iy* (conferring aminoglycoside resistance) was harbored by 19.4% of isolates, predominantly in *Salmonella* Typhimurium and *Salmonella* Goldcoast. The plasmid-encoded aminoglycoside phosphotransferase gene *APH-(4)-Ia* (linked to aminoglycoside resistance) was present in 9.7% of isolates. The multidrug and toxic compound extrusion (MATE) transporter gene *mdtK*, which confers resistance to fluoroquinolones, doxorubicin, and acriflavine, and the cell division regulator gene *sdiA*, a positive regulator of the AcrAB efflux pump associated with resistance to cephalosporins, fluoroquinolones, and phenicols, each exhibited a 6.5% carriage rate. Additionally, the plasmid-encoded aminoglycoside acetyltransferase gene *aac-(3)-IV* (mediating aminoglycoside resistance) was detected in 3.2% of isolates. Notably, no high-prevalence DR genes were detected across all serovars, and genes encoding resistance to sulfonamides, tetracyclines, or beta-lactams (e.g., *sul1*, *tet(A)*, *bla*_TEM-1_) were absent in these isolates. These findings indicate that while the strains retain conserved virulence traits supporting intestinal colonization, they harbor a limited repertoire of DR genes and lack extensive multidrug resistance (MDR) profiles.

Figure 1C illustrates the distribution of effector delivery system-related virulence genes among the 31 isolates (Table S3). Core T3SS genes from SPI-1 and SPI-2 were widely carried across strains, with serogroup D1 isolates showing the highest completeness of effector gene profiles (41.9%), followed by serogroup B strains (19.4%). Serogroups E1 and C2–C3 exhibited more diverse gene carriage patterns, accounting for 3.2–9.7% of isolates each. The overall distribution of these virulence genes was consistent with the serotype background of the strains.

### 3.3. Genomic Relatedness and Core-Genome Phylogeny

**Figure 2:**
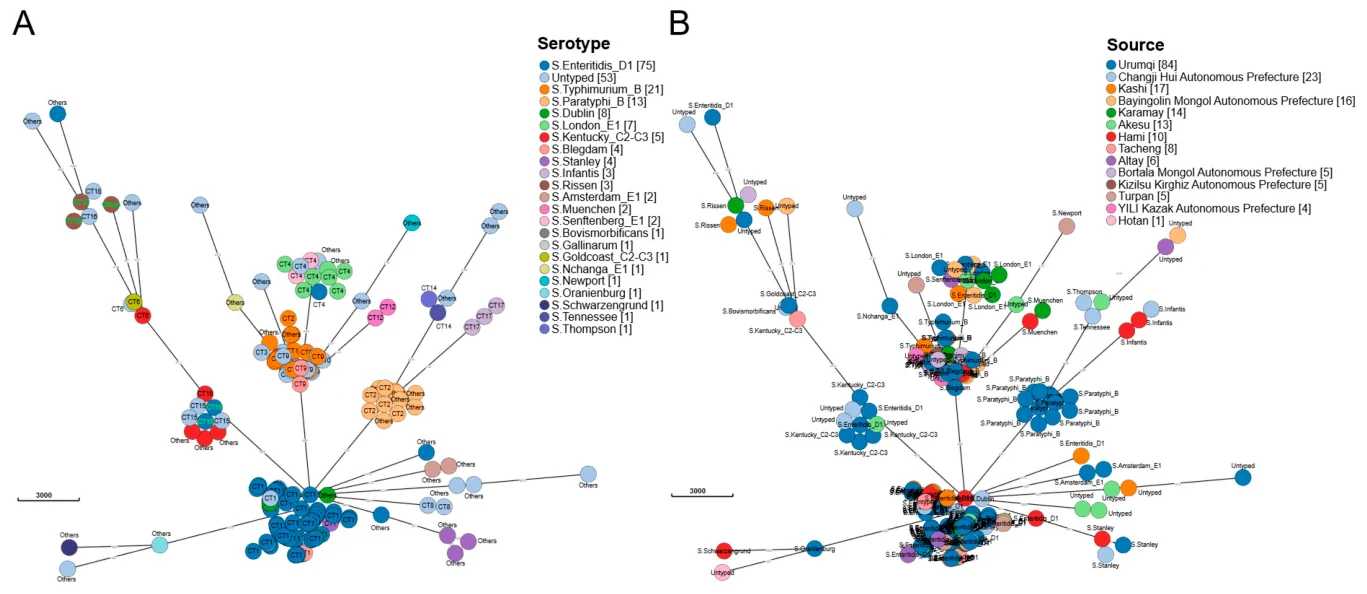
Distribution of serovars and geographic origins of *Salmonella* isolates included in clustering and phylogenetic tracing analyses. (**A**) Minimum Spanning Tree depicting the serovars distribution of isolates. Nodes represent individual isolates, with clustering patterns reflecting phylogenetic relatedness. The legend indicates serovars, with the number of corresponding isolates in parentheses. CT1–CT18 represents 18 clusters formed based on genomic clustering. (**B**) Minimum Spanning Tree illustrating the geographic origin distribution of isolates. Nodes represent individual isolates, with clustering patterns indicating regional transmission dynamics. Geographic origins (regions in Xinjiang, China) are indicated, with the number of corresponding isolates in parentheses.

To further explore the phylogenetic relationships and transmission dynamics of these strains, we integrated genomic and typing data of the 31 Urumqi-derived isolates with 181 additional *Salmonella enterica* isolates from other regions of Xinjiang to perform clustering and phylogenetic tracing analyses. Results showed that the largest cluster, CT1, comprised *Salmonella* Enteritidis and *Salmonella* Dublin, while CT3, CT9, and CT13 were dominated by *Salmonella* Typhimurium and other *Salmonella* serovars (Figure 2A, Table S3). Additionally, we found that *Salmonella enterica* strains identified in Urumqi were present in nearly all transmission clusters, indicating that Urumqi-derived isolates share close genomic relatedness with strains from other prefectures and are widely distributed across the phylogenetic landscape of the available dataset (Figure 2B, Table S3). However, given the heavier sampling representation of Urumqi, this pattern alone does not establish whether Urumqi serves as a primary source of transmission across Xinjiang. Interestingly, we also identified an independent transmission cluster in Urumqi, comprising CT2 and related clusters, with fewer than **50** single-nucleotide polymorphism (SNP) differences across the broader grouping (the core CT2 cluster had <20 SNPs) (Tables S3 and S4). All strains in this cluster were isolated from healthy individuals and typed as *Salmonella* Paratyphi B.

### 3.4. Salmonella enterica in the Intestines of Healthy Individuals

**Figure 3:**
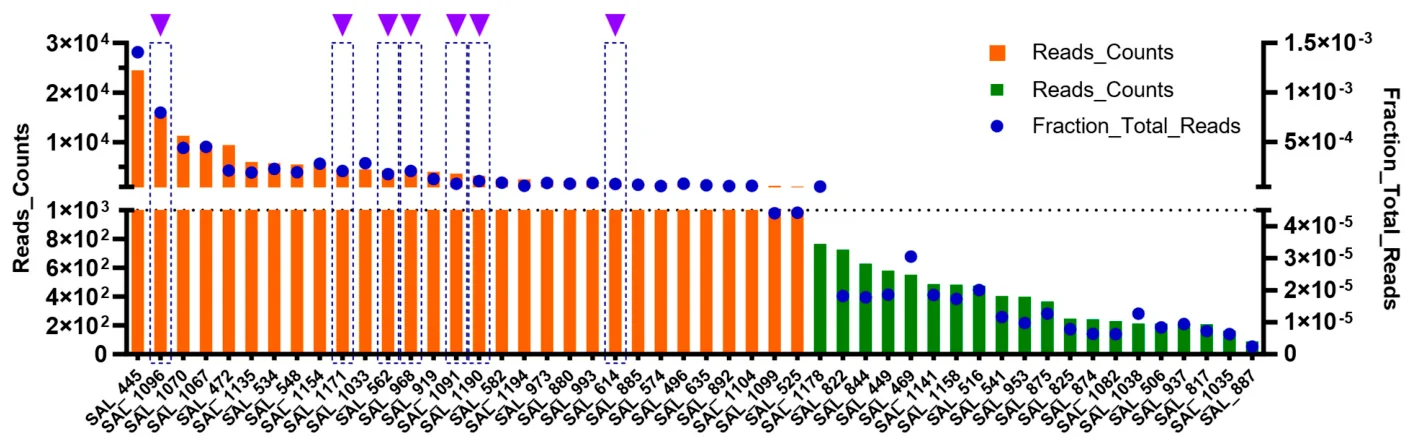
Distribution of *Salmonella enterica*-specific reads and their relative abundance in fecal samples from healthy individuals. The left y-axis represents the absolute count of *Salmonella*-specific reads (Reads_Counts), ranging from 0 to 3 × 10^4^, with a reference threshold of 1 × 10^3^ reads indicated. The right y-axis represents the fraction of *Salmonella*-specific reads relative to total sequencing reads (Fraction_Total_Reads), spanning from 0 to 1.5 × 10^−3^. Data points correspond to individual samples; the distribution of Reads_Counts and Fraction_Total_Reads facilitates the distinction between true *Salmonella* colonization and background sequence noise. The sample shown by the dashed box and purple inverted triangle represents an assembled sample identified by BLAST as containing the salmonella sequence that is longer than 1000 bp.

To assess *Salmonella enterica* colonization in 50 culture-negative healthy individuals, metagenomic sequencing was performed to quantify *Salmonella*-specific reads and relative abundance (Figure 3). A read-count cutoff was first established to distinguish putative *Salmonella*-specific sequence detected samples from background noise. Sensitivity analysis across alternative thresholds revealed a clear plateau in the estimated detection rate between 800 and 1000 reads, with core findings remaining consistent across the 800–1500 read range (Figure S1). The 1000-read cutoff was selected accordingly. Using this read-based criterion, 30 of 50 samples (60%) were positive. Using this read-based criterion, 30 of 50 samples (60%) were positive. The read-based positive group had significantly (*p* < 0.01) higher read counts (median ~12,000, range 8000–25,000) than the negative group (median ~400, all below threshold), with a corresponding median *Salmonella* read fraction of ~7 × 10^−4^ versus below 4 × 10^−5^ (Figure 3). All downstream comparative analyses were based on the read-based positive/negative grouping. Furthermore, to reduce false positives from short-read misclassification among closely related *Enterobacteriaceae* (e.g., *Salmonella*, *E. coli*, *Shigella*), an assembly-based verification step was applied. The samples with ≥1000 reads were subjected to de novo assembly, and only those with *Salmonella*-derived contigs > 1 kb were classified as assembly-verified positive. Using this combined criterion, 6 of 50 samples (12%) were confirmed positive (Table S6).

### 3.5. Impact of Carrying Salmonella Enterica on the Diversity and Community Structure of the Intestinal Microbiota in Healthy Individuals

To investigate the relationship between intestinal microbial community α-diversity and pathogenic sequence detection in 50 healthy individuals, we first established a comparative framework by characterizing diversity profiles across multiple grouping strategies. When samples were stratified by read-based detection of *Salmonella enterica*, *Shigella*, or *Klebsiella pneumoniae*, no significant differences were observed in any bacterial or archaeal α-diversity indices between the read-based positive and read-based negative groups. This absence of association extended to viral communities. Even when organized by combined *Salmonella enterica* and *Shigella* read-based positive and negative group, viral α-diversity indices remain statistically indistinguishable across groups, indicating that pathogenic sequence detection does not induce broad-spectrum diversity shifts in these microbial domains.

**Figure 4:**
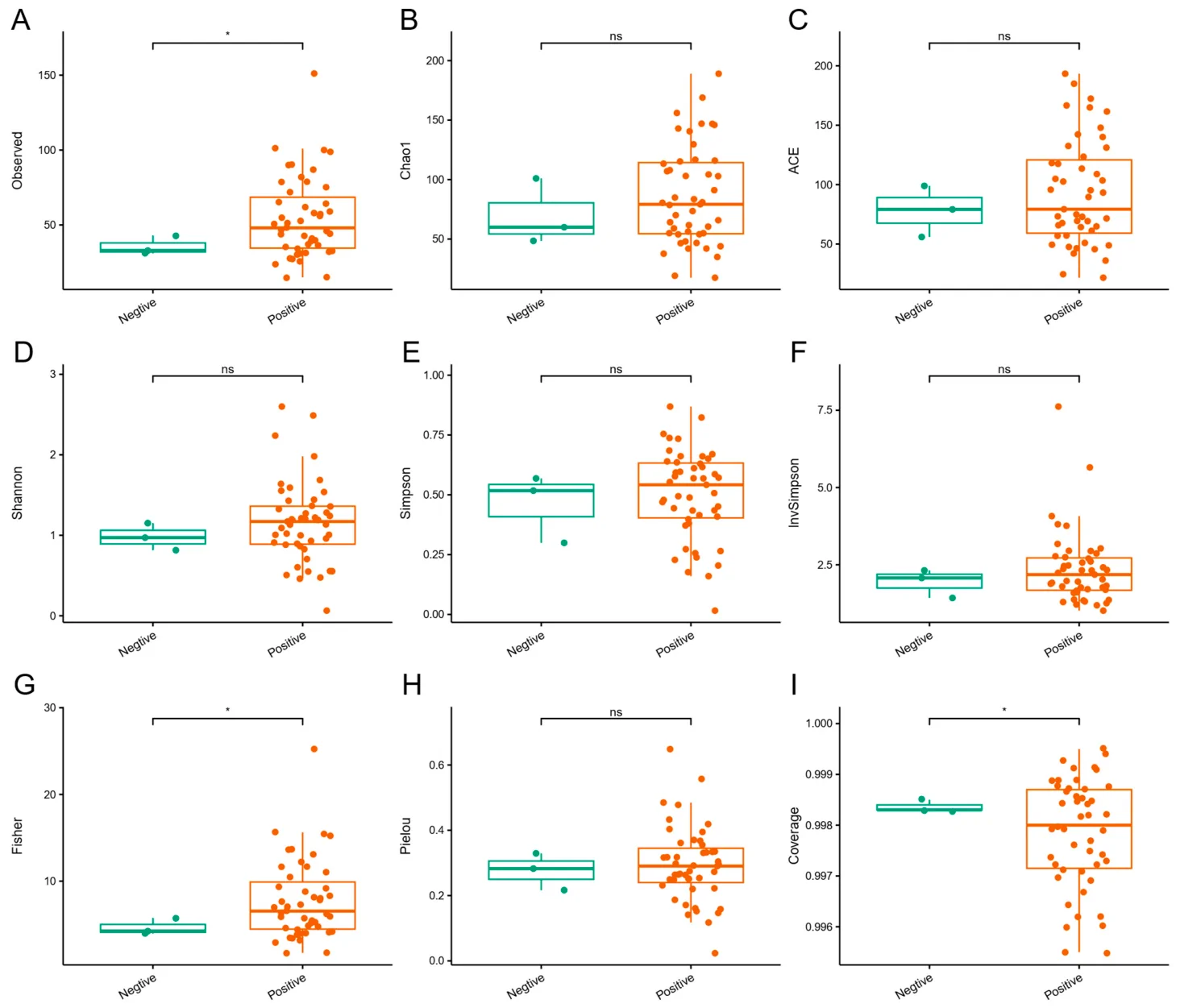
Boxplots of intestinal viral community α-diversity indices in 50 healthy individuals, stratified by *Klebsiella pneumoniae* specific sequence detection status. Samples were divided into two groups: *K. pneumoniae* read-based positive group (orange points) and read-based negative group (blue points). Each subplot (**A**–**I**) corresponds to a distinct α-diversity index: (**A**) Observed Species (actual number of distinct viral taxa detected), (**B**) Chao1 (estimated total species richness), (**C**) ACE (estimated taxonomic richness), (**D**) Shannon (integrates community richness and evenness), (**E**) Simpson (assesses community evenness), (**F**) InvSimpson (inverse Simpson index, reflecting community evenness), (**G**) Fisher (phylogenetic richness index), (**H**) Pielou (pure evenness metric), and (**I**) Goods Coverage (metric of sequencing depth adequacy). Boxplots represent the first (Q1) and third (Q3) quartiles, with the central line denoting the median; whiskers extend to data points within 1.5× the interquartile range (IQR), and points beyond this range are considered outliers. Statistical analyses were performed using the *t*-test: “ns” denotes no significant difference (*p* ≥ 0.05), “*” denotes significant difference (*p* < 0.05), while significant differences (*p* < 0.05) were observed for the Observed Species, Fisher, and Goods Coverage indices.

Focusing on viral communities stratified by *K. pneumoniae* read-based detection status, three key α-diversity indices differed significantly (Figure 4A,G,I), while Chao1, ACE, Shannon, Simpson, InvSimpson, and Pielou were non-significant (Figure 4B–F,H). The *K. pneumoniae* read-based positive group showed significantly higher Observed Species (*p* < 0.05, unpaired *t*-test), indicating greater viral taxonomic richness. The Fisher index was also significantly increased, supporting higher viral phylogenetic diversity. Conversely, Goods Coverage was significantly lower in the read-based positive group, suggesting a more complex viral community with more rare taxa. Overall, the stable *K. pneumoniae* read-based positive group is associated with targeted increases in viral richness and phylogenetic diversity, and reduced sequencing coverage, rather than global α-diversity changes.

**Figure 5:**
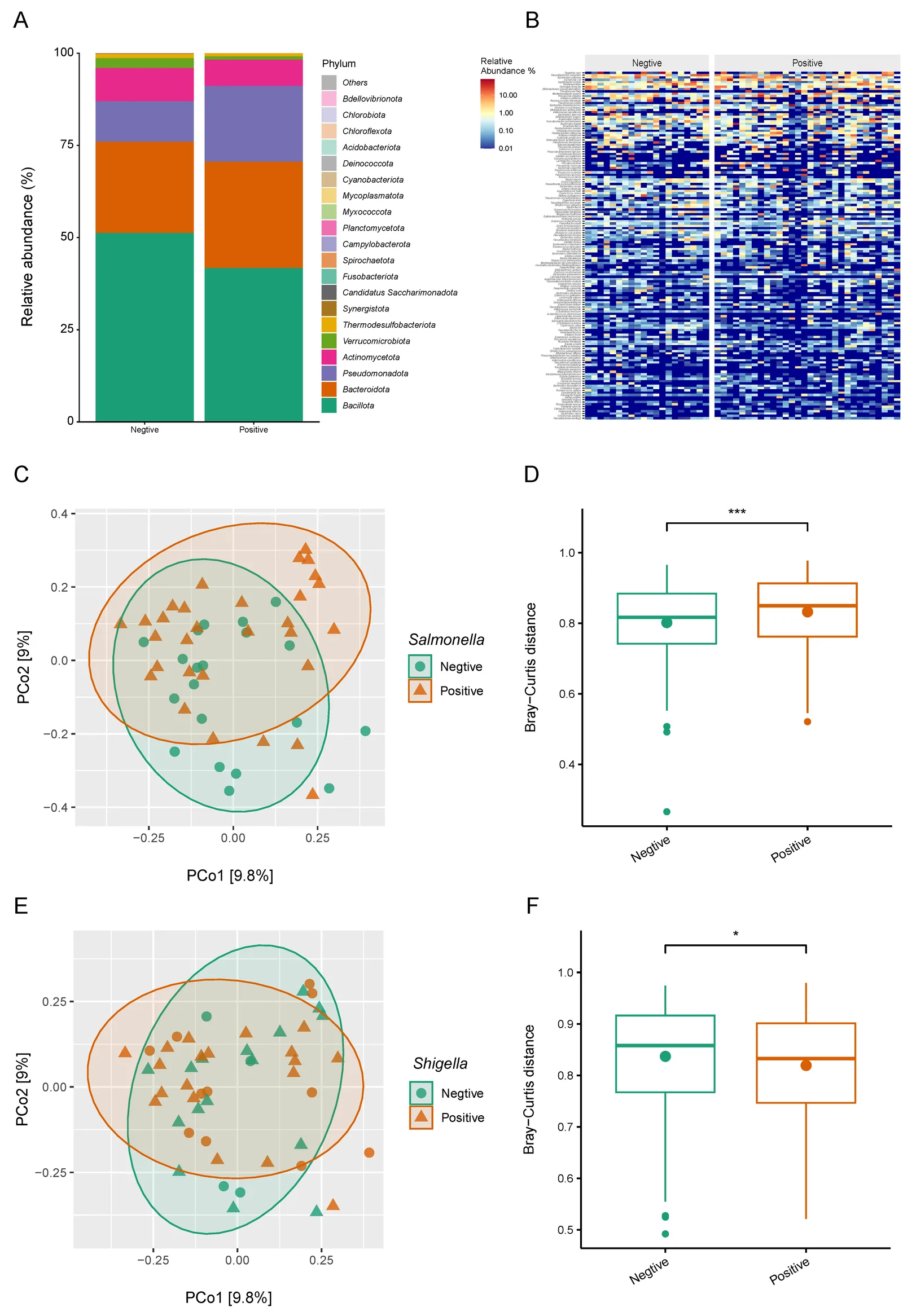
Intestinal bacterial community composition and β-diversity in 50 healthy individuals, stratified by *Salmonella*/*Shigella* specific sequence detection status. (**A**) Bar plot showing the relative abundance of dominant and minor phyla (%). (**B**) Heatmap of low-abundance phyla, with the y-axis displayed on a logarithmic scale for clarity. (**C**,**E**) Boxplots of inter-group Bray–Curtis distances for groupings by *Salmonella enterica* and *Shigella* read-based detection status, respectively. (**D**,**F**) Principal Coordinate Analysis (PCoA) plots based on Bray–Curtis distances for *Salmonella enterica* and *Shigella* read-based positive groups, respectively (PCo1 explained 9.8% of the variation, PCo2 explained 9%). PCoA showed no distinct clustering between groups, whereas ASV-level Bray–Curtis distances revealed significant compositional differences (*p* < 0.001 for *Salmonella*, *p* < 0.05 for *Shigella*; adonis test). Significance notations reflect adjusted *p* values. *, *p*_adj_ < 0.05; ***, *p*_adj_ < 0.001.

We further analyzed β-diversity and species composition (Figure 5). No significant differences were observed in phylum-level composition or overall β-diversity between *Salmonella* read-based positive and negative groups. At the phylum level (Figure 5A), dominant phyla (*Pseudomonadota*, *Bacteroidota*, *Bacillota*, *Actinomycetota*) and 18 minor phyla showed overlapping relative abundances with no significant variation. A species-level heatmap also showed no obvious distinction between groups (Figure 5B). PCoA confirmed no distinct community clustering by *Salmonella enterica* status, with extensive overlap and PCo1/PCo2 explaining 9.8% and 9% of total variation, respectively (Figure 5C). This limited variance explained by the first two axes means that PCoA captures only a fraction of the community variation; differences in higher-dimensional space may not be visible in this two-dimensional projection. Consistent with this, ASV-level community comparison using Bray–Curtis distances detected significant differences between the two groups in the full high-dimensional distance space (*p* < 0.001; adonis test) (Figure 5D). The betadisper test confirmed no significant difference in within-group dispersion between *Salmonella* read-based positive and negative groups (*p* > 0.05), indicating that the PERMANOVA result reflects true compositional differences rather than differences in group heterogeneity. Similarly, *Shigella* read-based positive and negative groups displayed identical trends in PCoA and community analyses (Figure 5E,F). Together, these results indicate that *Salmonella* or *Shigella* carriage is associated with subtle but statistically significant differences in intestinal microbial community composition, rather than a wholesale shift in community structure. Archaeal and viral communities showed similar patterns: no significant differences in PCoA, but significant divergence in ASV-level Bray–Curtis analyses (*p* < 0.05; adonis test) (Figures S2 and S3).

### 3.6. Analysis of Biomarkers and Co-Occurrence Networks

**Figure 6:**
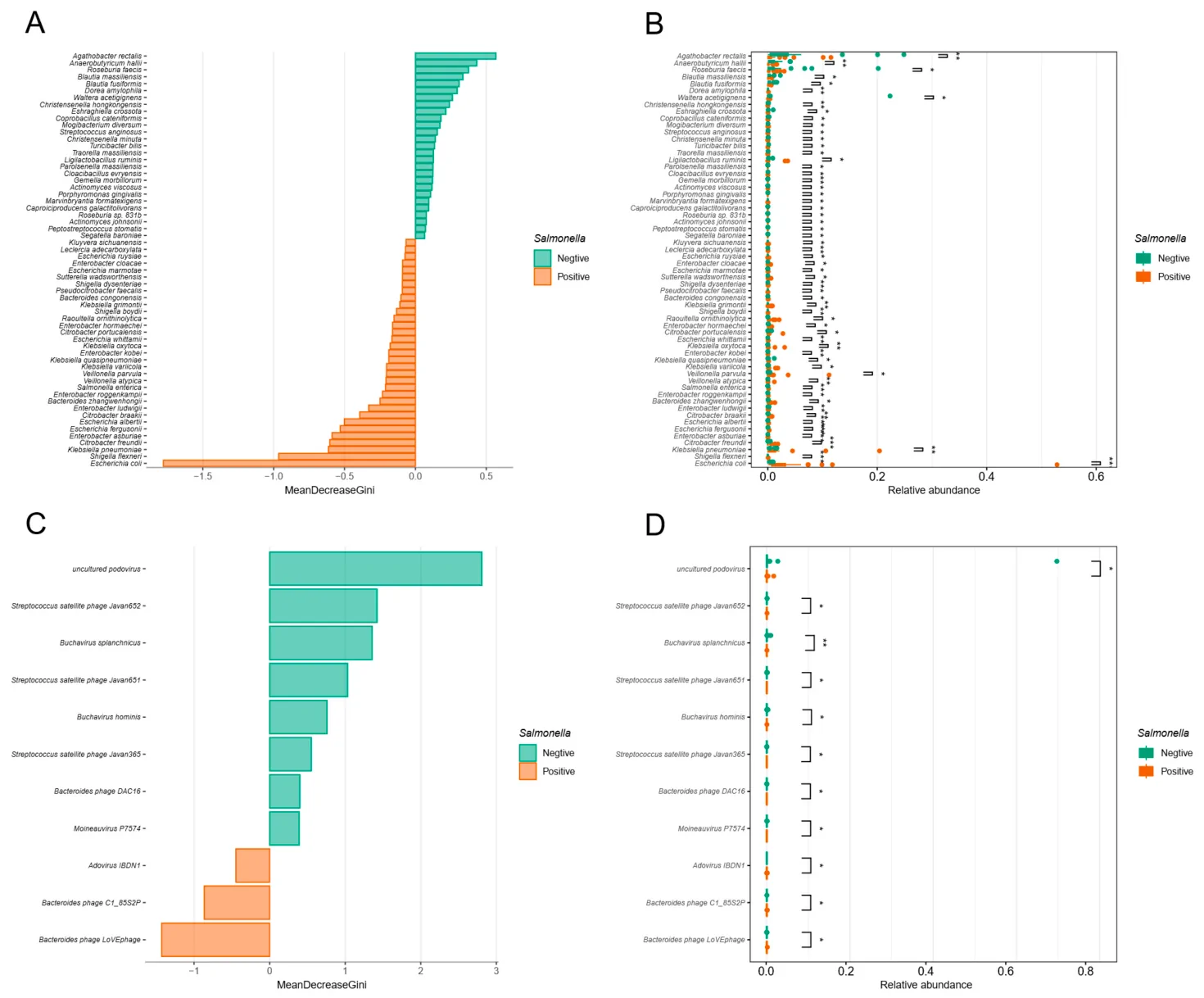
Random forest-based indicator species screening and relative abundance analysis of bacterial and viral taxa in 50 healthy individuals, stratified by *Salmonella enterica* specific sequence detection status. (**A**) Mean Decrease Gini ranking of bacterial taxa, with the x-axis representing discriminative importance for grouping by *Salmonella enterica* specific sequence detection status. (**B**) Heatmap showing the relative abundance of the top discriminative bacterial taxa identified in (**A**). (**C**) Mean Decrease Gini ranking of core viral indicator taxa. (**D**) Boxplots of the relative abundance of core viral indicator taxa, where the x-axis denotes *Salmonella enterica*-specific sequence-detection status and the y-axis represents relative abundance. All *p* values were adjusted using the Benjamini–Hochberg false discovery rate (FDR) method. Significance notations reflect adjusted *p* values. *, *p*_adj_ < 0.05; **, *p*_adj_ < 0.01.

To identify species-level microbial biomarkers linked to *Salmonella enterica* read-based detection status, we analyzed bacterial, archaeal, and viral communities from 50 healthy individuals using random forest analysis and *t*-tests. For the core bacterial community (Figure 6A,B), random forest analysis identified key discriminative species. Although the group with *Salmonella*-specific sequences did not significantly alter overall community structure, *Escherichia coli*, *Shigella flexneri*, and *Klebsiella pneumoniae* showed high abundances and significantly affected community structure, representing characteristic taxa associated with *Salmonella* read-based positive group. For the viral community (Figure 6C,D), six discriminative phage taxa were identified (e.g., *Bacteroides* phage DAC16, Moineauvirus P7574, *Adovirus* IBDN1), with no *Salmonella enterica*-specific phages included. *t*-tests confirmed significant abundance differences: *Bacteroides phage* DAC16 and *Adovirus* IBDN1 were more abundant in the *Salmonella* read-based positive group, while *Bacteroides* phage *LoVE* phage was less abundant. The random forest model achieved an overall accuracy of 90.23%, a sensitivity of 83.33%, a specificity of 91.17%, and an AUC of 0.8845 in 10-fold cross-validation, with the top discriminatory taxa listed in Table S7.

**Figure 7:**
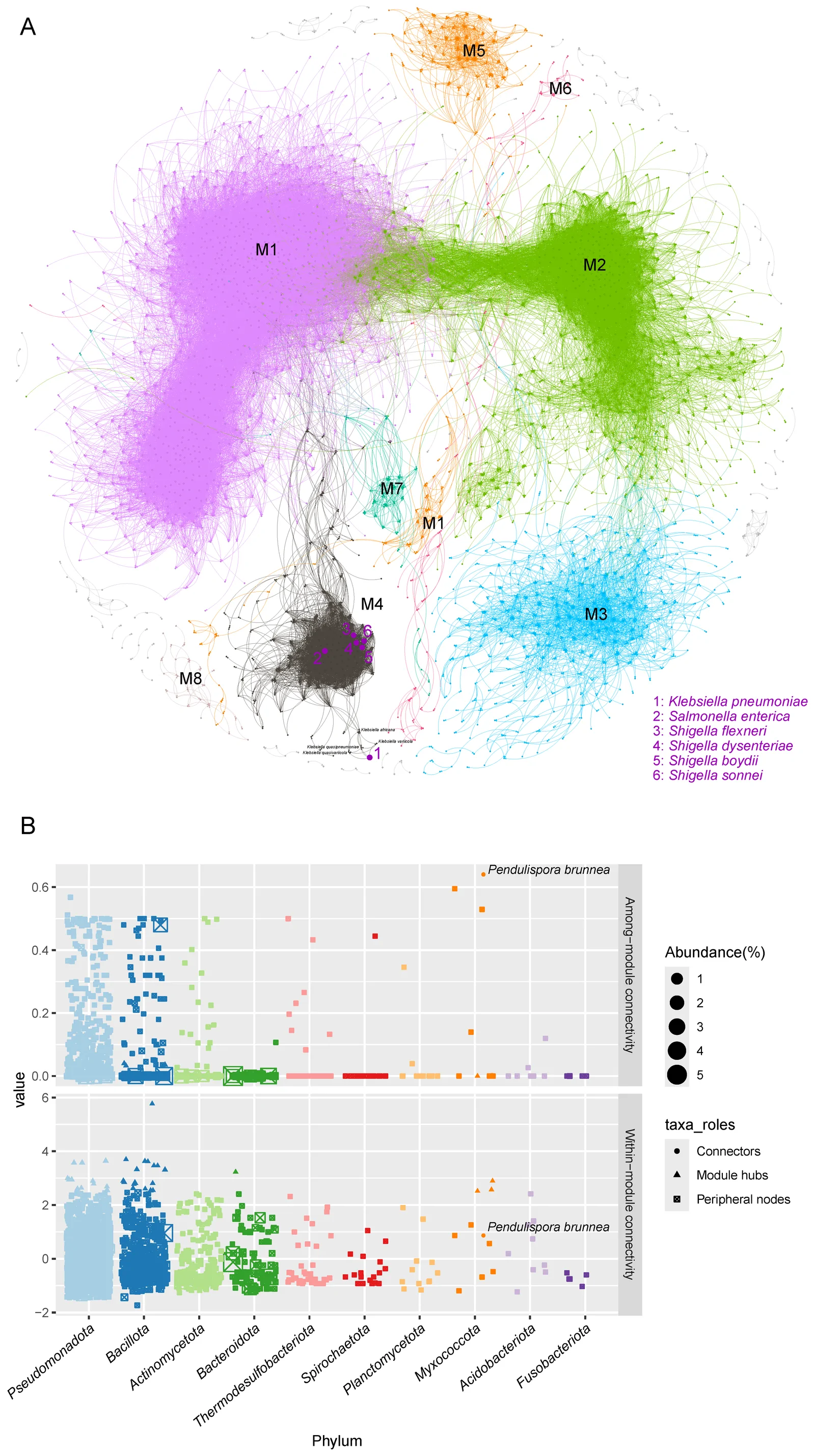
Microbial co-occurrence network and taxon network attribute analysis. (**A**) Microbial co-occurrence network. The nodes represent microbial taxa, clusters of different colors denote the eight largest co-occurrence modules (M1–M8) among the 54 modules identified, edges between nodes indicate significant pairwise associations among taxa, and target pathogenic taxa are labeled to show their distribution across modules. (**B**) Within-module and among-module connectivity of phylum-level taxa, reflecting a taxon’s centrality within its module and cross-module connectivity. Abundance data were integrated to classify taxa into ecological roles, including Connectors, Module hubs, and Peripheral nodes.

Finally, we performed microbial co-occurrence network analysis using Spearman correlations, RMT thresholding, and Louvain community detection (Figure 7) to assess the effects of target pathogens on the microbial network and identify associated taxa. The co-occurrence network identified 54 modules, the eight largest modules (M1–M8) are shown in the network visualization for clarity (Figure 7A). *Salmonella enterica*, *Klebsiella pneumoniae*, and *Shigella* all belonged to Module M4. *K. pneumoniae* showed strong correlations within *Klebsiella* and lay outside the module core. *Shigella* species (*S. flexneri*, *S. sonnei*, *S. boydii*) were at module edges with high intramodular connectivity. Enterobacteriaceae occupied the module center with strong internal connections.

The majority of taxa within Module M4, the module containing *Salmonella enterica*, *Klebsiella pneumoniae*, and *Shigella* species, belonged to the family *Enterobacteriaceae* (Table S5), indicating that target pathogens, especially *Salmonella*, occupy key ecological niches and closely interact with related species in the healthy gut. A hub node *Pendulispora brunnea* and other key taxa were identified (Figure 7B, Table S5), with the hub located in small Module M14 (only four taxa). Overall, the microbial network was relatively loose with highly independent modules.

## 4. Discussion

This study focused on Urumqi, Xinjiang. Using whole-genome and metagenomic sequencing, we systematically analyzed *Salmonella* carriage characteristics, genomic adaptability, and intestinal microbiota associations in healthy individuals, providing key data and theoretical support for the precise prevention and control of *Salmonella* infections across Xinjiang.

### 4.1. Public Health Implications of Salmonella Specific Sequence Detect Rates and Predominant Serovars in Healthy Populations

This study used metagenomic sequencing to investigate Salmonella detection in culture-negative healthy individuals in Urumqi. Initial read-based classification yielded a high apparent detection rate, but re-analysis with contig-level assembly verification substantially reduced this figure, as short reads from closely related *Enterobacteriaceae* (e.g., *E. coli*, *Shigella*) can be misclassified as *Salmonella* due to extensive sequence homology.

Despite this substantial reduction in false positives following assembly-based verification, the 12% *Salmonella*-positive rate by metagenomics remains markedly higher than the culture-confirmed carriage rate (31 isolates from >2000 individuals, ~1.5%). This discrepancy may be explained by two considerations. First, the detection of *Salmonella*-specific contigs in assembled sequencing data does not necessarily imply the presence of viable, culturable *Salmonella* cells in the original sample. The fragmented DNA from non-viable organisms or transiently ingested DNA may also be detected. Second, it is plausible that *Salmonella* is genuinely present in some culture-negative individuals but at very low abundance, suppressed by the commensal intestinal microbiota such that it falls below the detection limit of conventional culture methods. In this view, a negative culture result does not equate to the complete absence of *Salmonella*.

It is important to note that this metagenomic detection rate is not directly comparable to culture-based carriage rates reported in other studies, which typically range from 0.07% to 9.1% across different populations (e.g., 0.09% in Chinese catering workers [56], 0.07–0.113% in Japan [11], and 9.1% in Pakistani street vendors [57]). This high rate may be linked to Xinjiang’s diet rich in livestock and dairy products (major *Salmonella* reservoirs [58]) and Urumqi’s high population mobility. This rate was obtained by sensitive sequencing rather than culture, which can detect low-abundance *Salmonella enterica* sequences [59]. Such pathogens are suppressed by intestinal flora [60] and host immunity [61], without causing disease, but may trigger outbreaks once immunity or flora balance is impaired [61].

Serovars analysis showed that *Salmonella enterica* carried by healthy individuals was dominated by *Salmonella* Typhimurium and *Salmonella* Paratyphi B, together accounting for 60%, consistent with global NTS prevalence [2]. Both are pathogenic serovars: *Salmonella* Typhimurium causes acute gastroenteritis, while *Salmonella* Paratyphi B can induce paratyphoid fever and severe sepsis [2]. These strains harbored core T3SS genes (*invA*, *ssrA*) from SPI-1/SPI-2 and adhesion gene *fimA*, consistent with the genomic capacity for intestinal colonization [62]. The public health implications of asymptomatic carriage warrant further epidemiological investigation.

### 4.2. Genomic Adaptation Mechanisms of Asymptomatic Salmonella Enterica Carriage

Genome analysis showed that the *Salmonella enterica* strains from healthy individuals had a genome size of 4.51–4.95 Mb and GC content of 52.11–52.29%, highly consistent with reference strains (GCF_000006945.2) [63], indicating stable genome structure without large indels. Virulence gene analysis displayed conserved core genes and serovar-specific differentiation. All strains carried core SPI-1/SPI-2 T3SS and SPI-6 T6SS genes annotated as being associated with invasion, *Salmonella*-containing vacuole (SCV) formation [3], bacterial competition and adaptation [64], plus adhesion genes (*fimA*, *csgA*) and stress genes (*sodCI*) consistent with genomic capacity for colonization. No high-virulence exotoxin genes such as *cdtB* and *spvB* were detected [65], a genomic feature that is compatible with the asymptomatic carriage observed in this cohort. Whether this gene-content pattern underlies the asymptomatic phenotype requires functional validation.

Drug resistance gene analysis showed that the carriage rate of resistance genes in *Salmonella enterica* from healthy individuals was low (3.2–19.4%), with no multidrug-resistant (MDR) strains detected. Only a few resistance genes for aminoglycosides (*aac-(6*′)-*Iy*, *aph-(4)-Ia*) and fluoroquinolones (*mdtK*) were found; no genes for sulfonamides, tetracyclines, or β-lactams (e.g., *sul1*, *tet(A)*, *bla*_TEM-1_) were present, which is distinct from the global rising trend in *Salmonella enterica* drug resistance [4]. MDR strains resistant to ampicillin, chloramphenicol, and others have become widespread worldwide [66]. The low resistance in this study may be attributed to strict antibiotic control in Xinjiang, including limited misuse in livestock and less clinical antibiotic exposure in healthy individuals. The serovar-specific distribution of resistance genes (e.g., *aac(6*′)-*Iy* in *Salmonella* Typhimurium and *Salmonella* Goldcoast) is consistent with differential gene acquisition among serovars and highlights the need for targeted resistance surveillance in dominant serovars. However, these genomic findings should be interpreted cautiously: the inference of horizontal gene transfer remains unconfirmed without mobile genetic element and genomic context analyses, genotypic AMR prediction does not replace phenotypic susceptibility testing, and clinical treatment decisions should be guided by culture-based drug susceptibility results. Future studies incorporating phenotypic validation and MGE analysis are warranted.

### 4.3. Transmission Characteristics and Regional Diffusion Patterns of Salmonella enteria in the Urumqi Area

Phylogenetic analysis showed that Urumqi *Salmonella* strains distributed across multiple transmission clusters (CT1, CT3, CT9, CT13, etc.) and overlapped with nearly all clusters from other Xinjiang regions. This pattern is consistent with active inter-regional circulation of *S. enterica*, but should be interpreted cautiously. The overrepresentation of Urumqi isolates in the dataset may partly account for their broad distribution across clusters, and the current data cannot definitively establish Urumqi as a primary source or core hub of transmission across Xinjiang. As the political, economic, and transportation hub with frequent population mobility and intensive livestock and agricultural trade, Urumqi may facilitate inter-regional circulation of *Salmonella*, though the current uneven sampling does not allow definitive identification of a transmission hub. An independent local transmission cluster was also identified, with intra-cluster SNP differences < 50 (core CT2 cluster < 20 SNPs) and all typed as *Salmonella* Paratyphi B, suggesting an Urumqi-specific chain may be linked to local transmission dynamics, requiring further epidemiological tracing. However, several limitations preclude definitive source attribution: Urumqi was sampled more extensively than other prefectures, collection dates were incomplete for some isolates, and no epidemiological linkage data were available. Therefore, these findings should be interpreted as evidence of genomic relatedness and inter-regional circulation rather than a confirmed source–sink or core–radiation transmission pattern. A larger, geographically representative cohort with complete temporal and epidemiological metadata is needed to fully characterize transmission dynamics across Xinjiang.

### 4.4. Impact of Salmonella on the Intestinal Flora of Healthy Individuals

This study found that *Salmonella* did not significantly affect intestinal bacterial and archaeal α-diversity in healthy individuals, with only community composition differences observed at the ASV level. This is consistent with most pathogen–microbiome studies. Asymptomatic pathogens maintain a symbiotic balance with the host microbiota without causing large-scale disruption [67]. Random forest analysis identified *Escherichia coli*, *Shigella flexneri*, and *Klebsiella pneumoniae* (all Enterobacteriaceae) as signature taxa in the *Salmonella* read-based positive group, implying ecological niche synergy within the same family. These facultative anaerobes may share intestinal microenvironments and regulate each other via quorum sensing [19]. Additionally, *K. pneumoniae* significantly altered viral α-diversity (increased Observed Species and Fisher index, reduced Goods Coverage), indicating pathogen-specific effects on the microbiota. *K. pneumoniae* may indirectly modulate *Salmonella* colonization through viral community shifts; the exact mechanism requires further in vitro co-culture verification.

Co-occurrence network analysis showed that *Salmonella enterica*, *Klebsiella pneumoniae*, and *Shigella* all belong to module M4 and are closely associated with Enterobacteriaceae, suggesting a potential ecological association rather than demonstrating a specific intestinal niche or dependency. A hub node *Pendulispora brunnea* was identified in independent module M14 and linked to multiple core species in M4, suggesting it may act as a keystone taxon in the co-occurrence network of *Salmonella*-related microbial interactions. *P. brunnea* is a newly described myxobacterium (2024) in *Pendulisporaceae*, one of three species in the genus [68]. As a predatory bacterium, its predatory lifestyle may explain its association with multiple modules. Although as a predatory bacterium, its predatory lifestyle may explain its association with multiple modules, as a transient microorganism from diet and environment, there is insufficient evidence of its successful colonization in the human intestinal tract. Therefore, the discussion of its key link nodes still requires a large amount of supporting evidence. Although this study analyzed *salmonella enterica* and other pathogens in healthy Urumqi individuals, several limitations exist. First, the small sample size (31 strains, 50 metagenomic samples) and exclusive Urumqi origin cannot fully represent Xinjiang-wide carriage. The future work should expand samples to include Kashgar, Yili, Altay, and other regions. Second, lacking epidemiological data (age, gender, occupation, diet) prevented identification of specific drivers for the specific sequence detected rate. Third, no functional metabolic pathway analysis was performed to clarify intestinal metabolic impacts. Future plans include expanding sampling, integrating epidemiological surveys to define high-risk groups and transmission routes, verifying *Salmonella*–*E. coli*/*K. pneumoniae* interactions via co-culture, and establishing a microbiota-based screening model for precise prevention and control.

## 5. Conclusions

This study provides preliminary genomic and metagenomic evidence that asymptomatic *Salmonella* carriage occurs in healthy individuals in Xinjiang, with carried strains retaining intestinal colonization-associated virulence genes while showing low antimicrobial resistance. The co-occurrence of *Salmonella* with Enterobacteriaceae suggests an ecological niche within the gut microbiota that may facilitate persistence without disrupting overall community diversity. These findings have implications for public health surveillance: asymptomatic carriers may represent an underrecognized reservoir, and genomic surveillance integrating both culture and metagenomic approaches could improve detection of low-abundance carriage. The observed inter-regional genomic overlap further underscores the need for expanded geographically balanced sampling across Xinjiang to clarify transmission networks. Future studies incorporating larger cohorts, phenotypic validation, and longitudinal sampling will be essential to translate these genomic observations into targeted prevention and control strategies.

## Data Availability

The data that supports the findings of this study are openly available in NCBI at https://www.ncbi.nlm.nih.gov/bioproject/PRJNA1401443 (accessed on 30 August 2026), reference number PRJNA1401443.

## Supplementary Materials

The following supporting information can be downloaded at: https://www.mdpi.com/article/10.3390/pathogens15090930/s1, Figure S1: Changes in *Salmonella* carriage rate across different read count cutoffs; Figure S2: Intestinal archaeal β-diversity of 50 healthy individuals stratified by *Salmonella*, *Klebsiella*, and *Shigella* carriage status; Figure S3. Intestinal viral β-diversity of 50 healthy individuals stratified by *Salmonella*, *Klebsiella*, and *Shigella* carriage status; Table S1: The details of drug-resistant genes of 31 strain *Salmonella enterica* from healthy individuals; Table S2: Sample information of 2000+ healthy individual with *Salmonella enterica* isolates and metagenomic sequencing; Table S3: The clusters and serotype of 212 strain *Salmonella enteritidis*; Table S4: The differential SNPs in an independent clusters of S.Paratyphi_B; Table S5: The roles and parameters of network nodes; Table S6: The information of *Salmonella* reads and assembly identification in each sample; Table S7: Random forest feature importance of discriminatory bacterial taxa between *Salmonella*-specific sequence detected group and *Salmonella*-specific sequence undetected group; Table S8: Genomic characteristics of 31 healthy-associated *Salmonella enterica* isolates.

## Abbreviations

*ACE*	Abundance-based coverage estimator
*AMR*	Antimicrobial resistance
*ARO*	Antibiotic resistance ontology
*ARGs*	Antibiotic resistance genes
*ASV*	Amplicon sequence variants
*CARD*	Comprehensive antibiotic resistance database
*IQR*	Interquartile range
*MAGs*	Metagenome assembled genomes
*MDR*	Multidrug-resistant
*NTS*	Nontyphoidal serovars
*PCoA*	Principal coordinates analysis
*RES*	Reticuloendothelial system
*SCVs*	Salmonella-containing vacuole
*SPI*	Salmonella pathogenicity islands
*RGI*	Resistance gene identifier
*WGS*	Whole-genome sequencing
*WMS*	Whole metagenome sequencing
*XLD*	Xylose lysine deoxycholates
