# Supplementary material for: Whole-Genome Sequencing and Intestinal Metagenome Sequencing Revealed the Carriage and Transmission of Salmonella enterica in Xinjiang, China
Source: Pathogens. 2026 Sep 3;15(9):930. doi: 10.3390/pathogens15090930 (PMC13610005; doi:10.3390/pathogens15090930)
Supplement: Supplementary file 1 [file pathogens-15-00930-s001.zip › pathogens-4443647-Supplementary-Information.pdf]

Whole-genome sequencing and intestinal metagenome sequencing revealed the carriage and transmission of *Salmonella enterica* in Xinjiang, China

Authors:

Jia Huang[1†], Hongqing Zhao[2†], Fulong Wang[1], Beini Zeng[1], Zichao Ma[3], Rong Wang[1], Yang Yang[1], Muhetaer Amier[1], Bairena Yilihaer[4], Yaoqin Lu[1*]

[1] Institute of Pathogenic Biological Detection, Xinjiang Center for Disease Control and Prevention, Urumqi, China

[2] Administration Office, Institute of Infectious Disease Prevention and Control, Chinese Center for Disease Control and Prevention, Beijing, China

[3] Research and Technology Transfer Department, Xinjiang Center for Disease Control and Prevention, Urumqi, China

[4] Public Health College of Xinjiang Medical University, Urumqi, China

* Correspondence

Corresponding Author

lyq_superior@163.com

† These authors contributed equally to this work.

Keywords: Whole-genome sequencing; Metagenome sequencing; Gut; *Salmonella*; healthy people; Xinjiang, China.

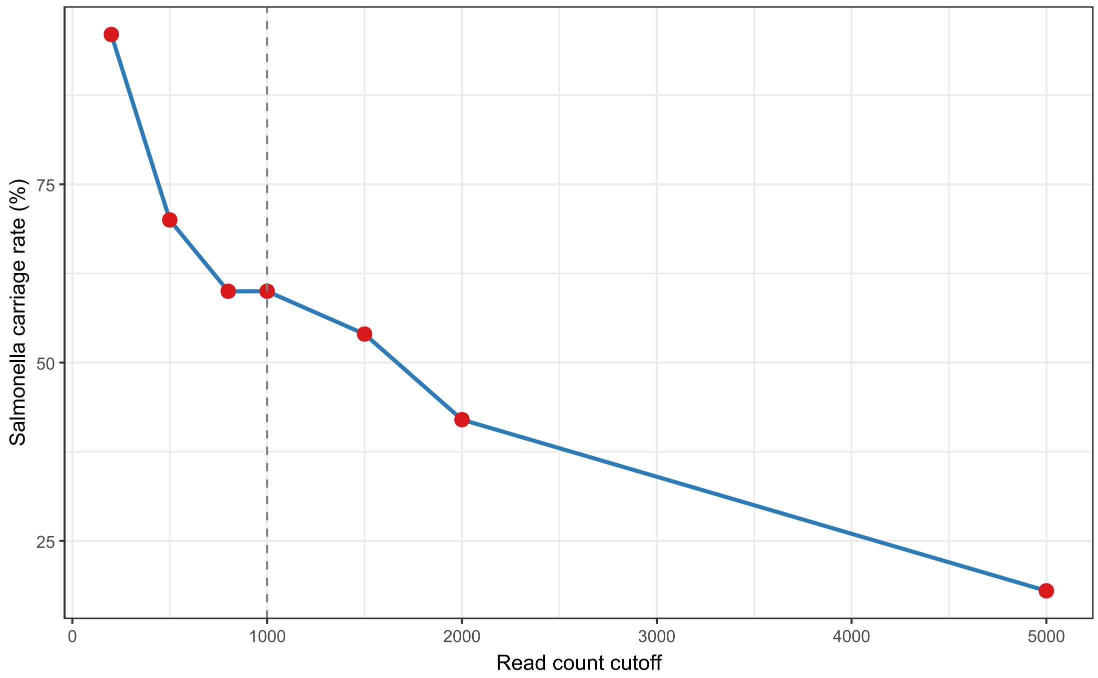

Figure S1. Changes in *Salmonella* carriage rate across different read count cutoffs. Sensitivity analysis shows the variation of estimated *Salmonella enterica* carriage rate under a gradient of read count thresholds. The vertical dashed line marks the 1000-read cutoff used in this study, which falls within the stable plateau of carriage rate.

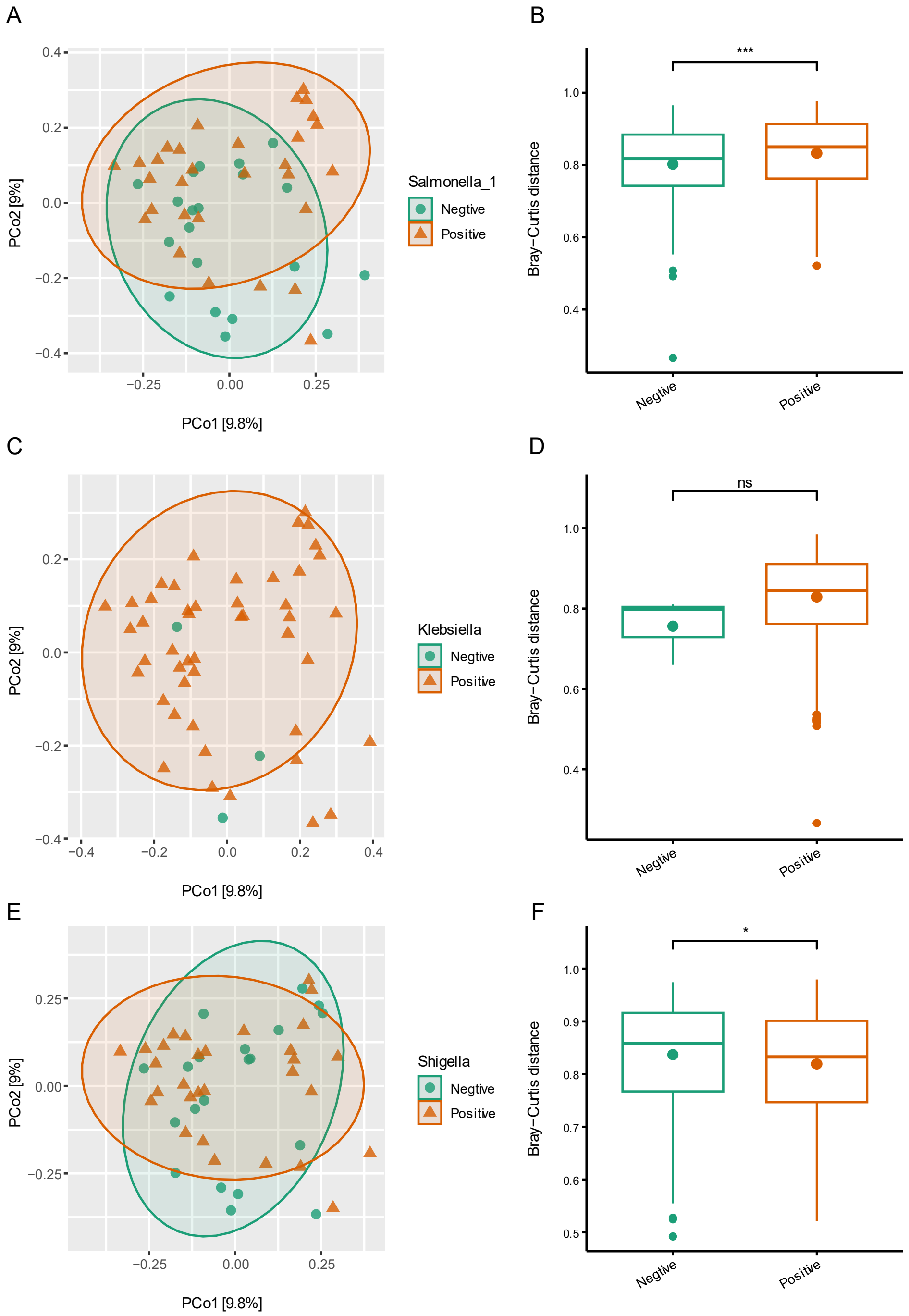

Figure S2. Intestinal archaeal β-diversity of 50 healthy individuals stratified by *Salmonella*, *Klebsiella*, and *Shigella* carriage status. (A, C, E) Boxplots of Bray-Curtis distances for *Salmonella*, *Klebsiella*, and *Shigella* groupings, respectively. (B, D, F) PCoA plots (Bray-Curtis) for *Salmonella*, *Klebsiella*, and *Shigella* groupings (PCo1=9.8%, PCo2=9%). PCoA showed no distinct clustering between groups, whereas ASV-level Bray-Curtis distances revealed significant compositional differences ($p < 0.0001$ for *Salmonella*, $p < 0.05$ for *Shigella*; adonis test).

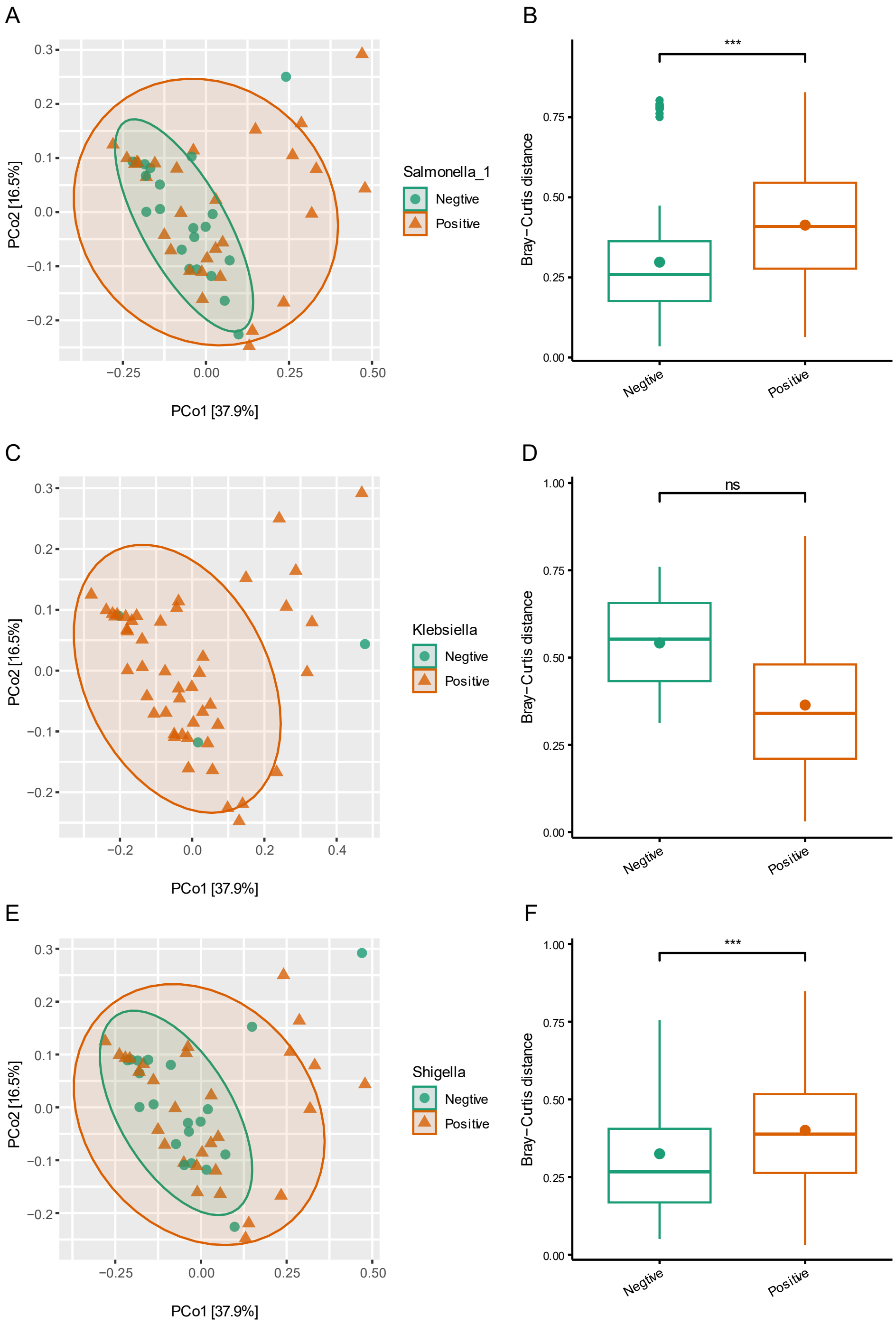

Figure S3. Intestinal viral β-diversity of 50 healthy individuals stratified by *Salmonella*, *Klebsiella*, and *Shigella* carriage status. (A, C, E) Boxplots of Bray-Curtis distances for *Salmonella*, *Klebsiella*, and *Shigella* groupings, respectively. (B, D, F) PCoA plots (Bray-Curtis) for *Salmonella*, *Klebsiella*, and *Shigella* groupings (PCo1=37.9%, PCo2=16.5%). PCoA showed no distinct clustering between groups, whereas ASV-level Bray-Curtis distances revealed significant compositional differences ($p < 0.0001$ for *Salmonella*, $p < 0.0001$ for *Shigella*; adonis test)
